# Marine ammonia-oxidising archaea and bacteria occupy distinct iron and copper niches

**DOI:** 10.1038/s43705-021-00001-7

**Published:** 2021-03-24

**Authors:** Roxana T. Shafiee, Poppy J. Diver, Joseph T. Snow, Qiong Zhang, Rosalind E. M. Rickaby

**Affiliations:** Department of Earth Sciences, University of Oxford, Oxfordshire, UK

**Keywords:** Biogeochemistry, Water microbiology

## Abstract

Ammonia oxidation by archaea and bacteria (AOA and AOB), is the first step of nitrification in the oceans. As AOA have an ammonium affinity 200-fold higher than AOB isolates, the chemical niche allowing AOB to persist in the oligotrophic ocean remains unclear. Here we show that marine isolates, *Nitrosopumilus maritimus* strain SCM1 (AOA) and *Nitrosococcus oceani* strain C-107 (AOB) have contrasting physiologies in response to the trace metals iron (Fe) and copper (Cu), holding potential implications for their niche separation in the oceans. A greater affinity for unchelated Fe may allow AOB to inhabit shallower, euphotic waters where ammonium supply is high, but competition for Fe is rife. In contrast to AOB, AOA isolates have a greater affinity and toxicity threshold for unchelated Cu providing additional explanation to the greater success of AOA in the marine environment where Cu availability can be highly variable. Using comparative genomics, we predict that the proteomic and metal transport basis giving rise to contrasting physiologies in isolates is widespread across phylogenetically diverse marine AOA and AOB that are not yet available in pure culture. Our results develop the testable hypothesis that ammonia oxidation may be limited by Cu in large tracts of the open ocean and suggest a relatively earlier emergence of AOB than AOA when considered in the context of evolving trace metal availabilities over geologic time.

## Introduction

Ammonia-oxidising archaea (AOA) and bacteria (AOB) mediate the first step of nitrification, the stepwise oxidation of ammonium (NH_4_^+^) to nitrite (NO_2_^−^) and nitrate (NO_3_^−^)—the central component of biogeochemical nitrogen cycling. A third group of aerobic ammonia oxidisers has also been identified, mediating complete aerobic ammonia oxidation to nitrate (comammox).^1^ AOA widely outnumber their bacterial counterparts in a range of environments including soils,^2^ freshwater streams and lakes^3–5^ and the marine environment.^6–12^ In the oligotrophic open ocean, archaeal *amoA* genes, an indicator of cell abundance, are up to three orders of magnitude greater than those of AOB,^7,10–13^ hypothesised to be due to the greater affinity of AOA for their shared substrate NH_4_^+^.^14^ Yet NH_4_^+^ growth kinetics are unable to solely account for the dominance of AOA in some coastal, estuarine and openocean environments^15–17^ where NH_4_^+^ supply is enough to support AOB growth, indicating that additional adaptive mechanisms contribute to the greater success of AOA. Further, the coexistence of AOB with AOA in marine environments albeit at low abundances of AOB,^6–13^ suggests that AOB occupy a distinct chemical niche allowing them to persist despite a low affinity for NH_4_^+^.^14^

The genome sequence of the marine AOA isolate, *Nitrosopumilus maritimus* SCM1 revealed the presence of numerous copper-rich halo- and plastocyanin enzymes^18^ in lieu of Fe-dense cytochromes typically found in AOB^19,20^ alluding to a potential role for trace metals in the niche separation between AOA and AOB. Subsequent studies^21,22^ have revealed that the cupric ion (Cu^2+^) and unchelated Fe (Fe´) requirement for optimal growth in *N. maritimus* (1 pmol L^−1^ Cu^2+^ and 550 pmol L^−1^ Fe´) are above those typically measured in the marine environment (~0.01–0.1 pmol L^−1^ Cu^2+^ and ~1 pmol L^−1^ Fe), presenting the potential for marine nitrification to be limited by these metals in the open oceans. Growth limitation by Fe or Cu is welldocumented in other components of the marine N-cycle including nitrogen fixation,^23,24^ NO_3_^−^ assimilation^25^ and denitrification.^26^ There has yet to be an equivalent physiological analysis of marine AOB Fe and Cu requirements for growth, precluding an accurate assessment of the role of these metals in modulating marine ammonia oxidation rates and shaping the niche separation between AOA and AOB. Comparison of Fe and Cu requirements also provides a new perspective on the longstanding question of AOA and AOB relative emergence, drawing on the idea that extant microorganisms contain imprints of shifting trace metal bioavailabilities over geologic time at the cellular level.^27–30^

Here, we provide the first physiological examination of the trace metal requirements of any marine AOB isolate, reporting the free inorganic Fe and Cu requirements of *Nitrosococcus oceani* strain C-107 (ATCC 19707)*. N. oceani* belongs to the γ-subdivision of the Proteobacteria, an omnipresent AOB group in seawater.^31,32^ We compare the physiological response of *N. oceani* to unchelated Fe and Cu availability with that of the cultured marine AOA isolate, *Nitrosopumilus maritimus* strain SCM1 from previous work^21,22^ supplementing the published data with the intracellular Cu quota of *N. maritimus* SCM1. To explore whether the physiologies and uptake strategies of *N. oceani* C-107 and *N. maritimus* SCM1 reflect broader marine AOA and AOB communities, pure isolates for which are not yet available, we perform a two-pronged bioinformatic analysis of the metal-binding sites and metal transporter systems in AOA and AOB genomes.

## Methods

### *Nitrosococcus oceani* C-107 cultures and growth media

Polycarbonate culture vessels and culturing apparatus were acid-cleaned in 10% (v/v) TraceMetal™ grade HCl (Fisher Scientific, Loughborough, UK) for 24 h and UV-sterilised before use. Triplicate 10ml cultures of *Nitrosococcus oceani* strain C-107 (ATCC 19707 originally isolated from the open North Atlantic) herein referred to as *N. oceani*, were grown in modified SCM medium^33^ amended with additional NH_4_Cl (final NH_4_^+^ concentration 10mM) and maintained at 30 °C in the dark on a rotary shaker (150 r.p.m.). Basal salt medium and macronutrients were treated with Chelex-100 resin (BioRad, Watford, UK) to remove trace metal contaminants^34^ and passed through an acid-washed (10% HCl as before) 0.2µm polycarbonate filter to sterilise in a metal-free clean laboratory. The final pH of the media was 7.5 and was buffered with HEPES. To investigate the effect of unchelated inorganic Fe (Fe´) and copper (Cu´) concentrations on *N. oceani* growth, ﻿ethylenediaminetetraacetic acid (EDTA, ﻿Merck, Darmstadt, Germany) was used to buffer Fe, Cu and other metals in cultures. Fe´ and Cu´ were controlled by varying the addition of FeCl_3_ or CuCl_2_ from filter-sterilised concentrated stocks made using >99.999% trace metal basis salts (Sigma Aldrich, UK) and maintaining a constant concentration of EDTA at 25 µmol L^−1^ (Fe experiments) or 120 µmol L^−1^ (Cu experiments)—EDTA concentrations which have been shown to be non-toxic to *Nitrosococcus* in previous work.^35,36^ Fe´, Cu´ and cupric ion (Cu^2+^) concentrations were calculated using Visual Minteq software.^37^ Cu^2+^ is presented in main figures, in order to allow for comparison with previous Cu growth experiments of AOA and phytoplankton Cu studies and measurements made in the field, which use Cu^2+^. Cu´ values are given in Table 1 and Supplementary Fig. 1. Background Fe and Cu blank levels of 1.4 and 12.5 nmol L^−1^, respectively, from basal SCM medium, were measured using inductively-coupled plasma mass spectrometry (ICP-MS)^38^ and included in the calculation of metal speciation. Cultures were acclimated to Fe´ and Cu´/Cu^2+^ by transferring cultures consecutively during the late exponential growth phase into new media until growth rates did not vary with statistical significance (ANOVA, *P* < 0.05). The growth rate was determined by measuring nitrite (NO_2_^−^) spectrophotometrically over time as in previous studies (Supplementary Fig. 2,^33,39–41^), the production of which correlates with cell counts.^22,23^ Specific growth rate (d^−1^) was calculated over the linear phase of semi-log plots of nitrite (NO_2_^−^) concentration over time.

**Table 1:** Relationships among total and inorganic unchelated Fe (dFe and Fe´), total Cu, unchelated Cu and cupric ion (dCu, Cu´ and Cu^2+^), growth rate (μ), relative growth rate (u/μ_max_), Fe, Cu and P quota per cell and Cu and Fe normalised to cellular P, Fe´ steady-state uptake rate (*ρ*_ss_) and steady-state uptake rate normalised to Fe´ (*K*_*in*_).

dCu	dFe	Fe´	Cu´	Cu^2+^	u	u/μ_max_	Cell Fe	Cell P	Cell Cu	Fe:P	Cu:P	*ρ*_*ss*_	*K*_*in*_	K_*in*_/S.A.
nmol L^−1^	µmol L^−1^	pmol L^−1^	pmol L^−1^	pmol L^−1^	d^−1^		mol cell^−1^	mol cell^−1^	mol cell^−1^	mmol mol L^−1^	mmol mol L^−1^	mol Fe cell^−1^ hr^−1^	L cell^−1^ hr^−1^	
*N. oceani* C-107														
2.5	0.05	9	0.15	0.10	n.g.	0								
2.5	0.2	35	0.15	0.10	n.g.	0								
2.5	0.57	100	0.16	0.10	0.41 * (0.01)	0.66	1.6 × 10^−17^ (1.4 × 10^−18^)	3 × 10^−15^ (1.2 × 10^−15^)		6.1		2.8 × 10^−19^ (2.4 × 10^−20^)	2.8 x 10^−9^ (2.4 × 10^−10^)	2.2 × 10^−10^
2.5	1	179	0.16	0.11	0.41 * (0.01)	0.66								
2.5	2	376	0.16	0.11	0.46 ** (0.01)	0.78	3 × 10^−17^ (1.1 x 10^−17^)	3 × 10^−15^ (2.7 × 10^−15^)		10.9		6.2 × 10^−19^ (2.1 × 10^−19^)	1.7 × 10^−9^ (5.5 × 10^−10^)	1.3 × 10^−10^
2.5	4	825	0.16	0.10	0.52 ** (0.02)	0.85	4.1 × 10^−17^ (1.9 × 10^−17^)	2 × 10^−15^ (6.1 × 10^−16^)		19.8				
**2.5**	**13**	**5006**	**0.20**	**0.10**	0.61 *** (0.01)	**1.00**	**6.1** × **10**^**−17**^ **(1.1** × **10**^**−17**^**)**	**2.2 x 10**^**−15**^ **(3.6** × **10**^**−15**^**)**		**27.9**				
**2.5**	**17**	**10063**	**0.21**	**0.10**	0.54 *** (0.03)	**0.93**								
*N. oceani* C`107														
1.4	7.5	6500	0.07	0.05	n.g.	0.00								
2	7.5	6500	0.10	0.07	0.44 * (0.02)	0.72								
**2.5**	**7.5**	6500	**0.13**	**0.09**	0.61 ** (0.04)	**1.00**		**2.5** × **10**^**−15**^ **(1.7 x 10**^**−15**^**)**	**3.1** × **10**^**−19**^ **(1.9** × **10**^**−19**^**)**		**0.13**			
**4.9**	**7.5**	**6500**	**0.21**	**0.14**	0.55 ** (0.08)	**0.90**								
7.9	7.5	6501	0.40	0.27	0.52 *** (0.08)	0.85								
32.5	7.5	6505	1.68	1.01	0.43 † (0.05)	0.71								
68	7.5	6510	3.49	2.37	0.48 † (0.04)	0.78								
125	7.5	6519	6.89	4.68	0.48 † (0.07)	0.78								
680	7.5	6604	35.93	24.63	0.47 † (0.06)	0.77		6.8 × 10^−14^ (1.3 × 10^−14^)	6.9 × 10^−19^ (8.6 × 10^−19^)		**0.7**			
52.00	7.5	6734	78.84	53.54	0.44 † (0.05)	0.72								
*N. maritimus* SCM1														
**7** × **10**^**−8**^	**7500**	**7585**	**1.66**	**1.00**	**0.51 (0.02)**	**1.00**		**2.6** × **10**^**−15**^ **(1.3** × **10**^**−15**^**)**	**4.9** × **10**^**−18**^ **(1.9** × **10**^**−19**^**)**		**1.89**			

**Fig. 1:**
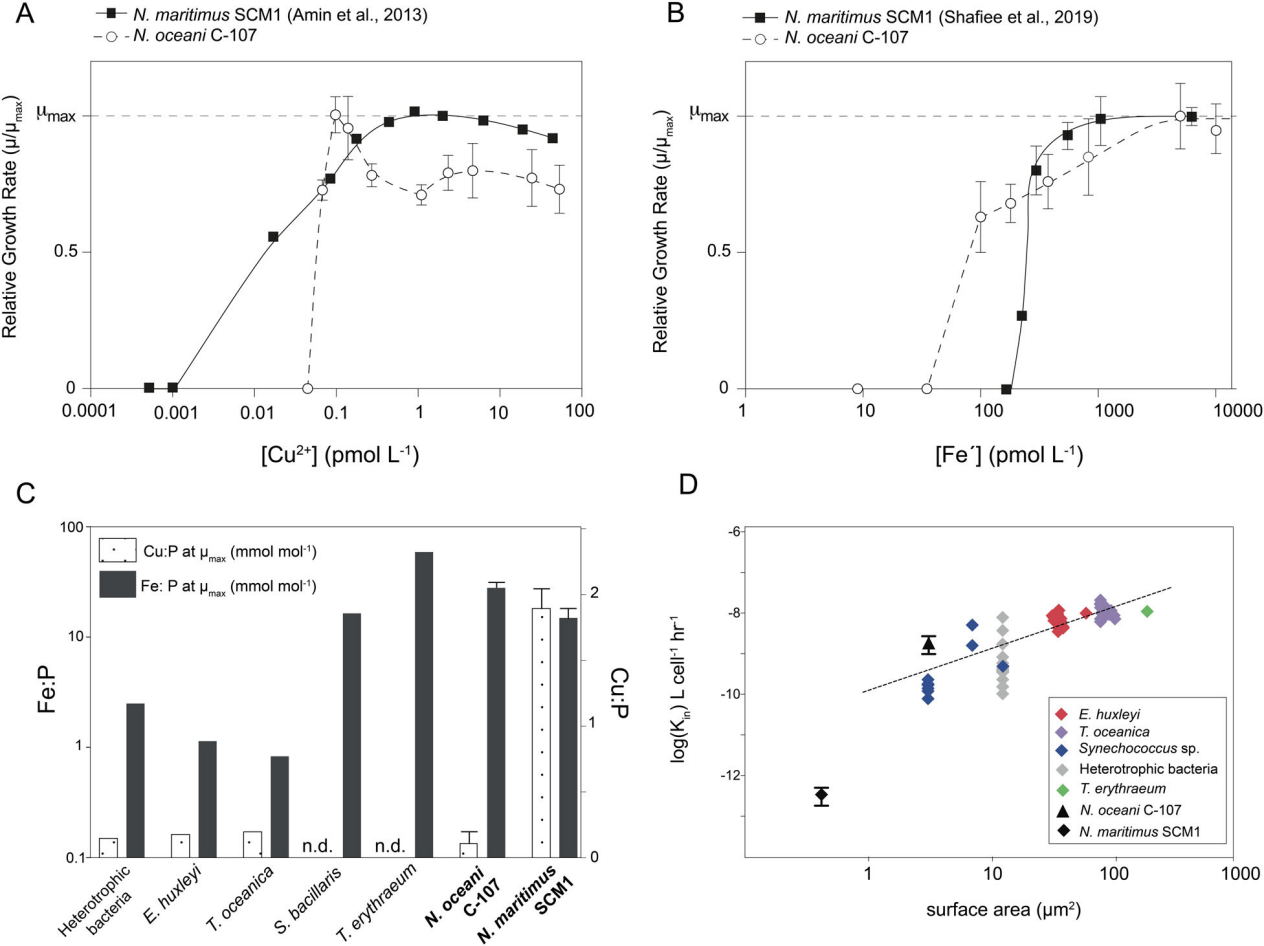
Iron and copper dose-response curves, cellular quotas and steady-state uptake rates of AOA and AOB isolates, *Nitrosopumilus maritimus* and *Nitrosococcus oceani*. Effect of (**A**) cupric ion concentration [Cu^2+^] (pmol L^−1^) (**B**) unchelated Fe concentration (Fe´) on the relative growth rate (μ/μ_max_) of the ammonia-oxidising bacterium *Nitrosococcus oceani* strain C-107 (open circles and dashed line) compared with previously published data on the ammonia-oxidising archaeon *Nitrosopumilus maritimus* strain SCM1^21,22^ (closed squares and solid black line—error bars within points on *N. maritimus* data). Error bars on *N. oceani* points represent standard deviation calculated on *n* = 21 (three replicates were conducted for each treatment in a total of seven acclimated sub-cultures—calculated to be statistically indistinguishable, ANOVA, *P* < 0.01). Error bars on *N. maritimus* SCM1 data points in 1B) represent standard deviation on *n* = 20, as calculated previously.^23^ (**C**) Intracellular Cu and Fe quota in mol cell^−1^ normalised to mol P cell^−1^ at maximum relative growth rate, μ_max_, in *N. oceani* and *N. maritimus* (Cu quota calculated in this study, Fe quota calculated in ref. ^22^) compared with published quota data of numerous marine phytoplankton (data and references provided in Supplementary Table). Fe:P and Cu:P at μ_max_ in *N. oceani* was measured at 5006 pmol L^−1^ Fe´ and 0.07 pmol L^−1^ Cu^2+^, respectively. Cu:P at μ_max_ in *N. maritimus* SCM1 was measured at 0.97 pmol L^−1^ Cu^2+^. Additional Fe and Cu quota under varying [Fe´] and [Cu^2+^] is provided in Table 1 and Supplementary Fig. 1. See Supplementary Materials for further discussion on the use of quota data at μ_max_. Error bars on *N. oceani* data represent standard deviation, *n* = 3. **D** log *K*_*in*_, Fe´ steady state-uptake constant, in L cell^−1^ h^−1^ as a function of cellular surface area (μm^3^) in *N. oceani* (filled diamond) compared with published data of *N. maritimus*^22^ and other marine phytoplankton.^48^ Error bars represent standard deviation, *n* = 6.

Values in brackets indicate standard deviation. Treatments in bold indicate the first instance that μ_max_ is reached, quotas for which are used for comparison in Fig. 1C. Treatments are grouped by the symbols *, **, *** and † - treatments with the same symbols are statistically indistinguishable from each other (*P* < 0.05, two-tailed *T* test or one-way ANOVA), but vary with statistical significance from other treatments (*P* < 0.05, two-tailed *T* test or one-way ANOVA).

### *Nitrosopumilus maritimus* SCM1 cultures

To supplement previously published data with the intracellular Cu quota of *Nitrosopumilus maritimus* strain SCM1 (herein referred to as *N. maritimus*) *N. maritimus* cultures were maintained as previously described.^22^ In brief, triplicate cultures of *N. maritimus* were grown in SCM media^33^ treated with Chelex-100 resin, with 1 mM NH_4_Cl (final concentration) and maintained in the dark at 28 °C. Cultures were acclimated to optimal Cu^2+^ concentrations (1 pmol L^−1^ Cu^2+^) determined in a previous study.^22^ Full details of *N. maritimus* culturing method are provided in Supplementary Materials.

### Trace metal quota analysis of cells

All samples and reagents were processed using trace metal clean techniques and acid-washed plastic wear in a clean laboratory before trace element analysis. *N. oceani* and *N. maritimus* cells at μ_max_ (see Supplementary Materials for full discussion of Fe´ and Cu^2+^ chosen for trace metal analysis) were harvested in mid-exponential phase by centrifugation at 5000×*g* for 25 min and then rinsed three times with Chelex-100-treated SCM media to remove weakly bound surface metals.^38^ Cells were digested in acid-cleaned Teflon as follows: refluxing at 100 °C with 16N quartz-distilled (q.d.) HNO_3_ (produced in-house) and H_2_O_2_ (ROMIL UpA™, Cambridge, UK) in a ratio of 3:2 overnight. The liquid was then allowed to evaporate to dryness and 2% q.d. HNO_3_ was added to reflux for an hour to resuspend the dried samples. ^56^Fe, ^31^P and ^63^Cu concentrations were determined using a Quadrupole ICP-MS Perkin Elmer NexION 350 with Elemental Scientific Flow Injection Auto-sampler (FIAS) in a method optimised for high-salt matrices.^38^

### Steady-state iron uptake rate

The steady-state Fe´ uptake rate, *ρ*_*ss*_, the product of intracellular Fe quota (mol cell^−1^) and specific growth rate (hr^−1^) and steady-state uptake constant, *K*_*in*_, *ρ*_*ss*_ normalised to Fe´ in the medium, were calculated for *N. oceani* as per previous work^22,42^ using Fe-limited cellular quotas (Table 1). Cellular quotas measured in *N. oceani* cells cultured at 100 pmol L^−1^ Fe´ (66% of μ_max_) and 376 pmol L^−1^ Fe´ (78% of μ_max_) were used in calculations. To obtain a steady-state uptake constant per unit surface area (S.A.), S.A. was calculated based on a spherical cell shape and using a radius of 1 μm obtained from SEM imaging (Supplementary Fig. 3), preparing cells using the same protocol as in ref. ^23^, which is fully outlined in Supplementary Materials.

### FeDFB and ferrozine experiments

To examine whether *N. oceani* can utilise organically chelated Fe, *N. oceani* was cultured in medium with the addition of the siderophore desferrioxamine B mesylate (DFB, Merck) to buffer Fe. *N. oceani* was cultured under high and low concentrations of total dissolved Fe, dFe, corresponding to 5006 pmol L^−1^ dFe and 100 pmol L^−1^ dFe. A 1.3-fold excess of DFB was added over Fe, in order to reduce Fe´ to negligible concentrations (<1 × 10^9^ pmol L^−1^ Fe´). EDTA was added in a low concentration (12 µmol L^−1^ EDTA) to buffer other trace metals. We used the ferrozine assay^42^ to identify whether *N. oceani* adopts a reductive Fe uptake pathway in Fe acquisition. Ferrozine (FZ, 5,6-diphenyl-3-(2-pyridyl)-1,2,4-triazine) binds with any available Fe^2+^ in the medium forming a Fe(II)–FZ_3_ complex which is unavailable for uptake across the outer cell membrane.^43^ Inhibition of growth with the addition of FZ therefore indicates that cells are actively reducing Fe^3+^ to Fe^2+^ during Fe acquisition. *N. oceani* cultures were acclimated to replete and deplete Fe´ (5006 pmol L^−1^ Fe´ and 100 pmol L^−1^ Fe´), then transferred to new media with 200 µmol L^−1^ FZ. Growth rate (μ) was determined as previously outlined.

### Bioinformatic predictions of AOA and AOB physiology in wider communities

The limited number of marine AOA and AOB species isolated into pure culture precludes an extensive physiological analysis of the trace metal requirements across the wider AOA/AOB community using culture-based approaches. To explore whether the physiologies of *N. oceani* C-107 and *N. maritimus* SCM1 may reflect broader marine AOA and AOB communities, we used available genome-predicted proteomes (downloaded from UniProt: https://www.uniprot.org) to annotate the hypothetical Cu- and Fe-binding sites in marine AOA and AOB, on the basis that physiological requirement typically scales with the number of metal-binding sites. In addition, we annotated the presence of metal transporter systems to explore whether the genetic basis for the contrasting metal affinities of *N. oceani* and *N. maritimus* in culture is ubiquitous among the broader AOA and AOB community.

For both analyses, only genome-predicted proteomes with a high genome completeness (>96%) were used (full search criteria outlined in Supplementary Materials). For annotation of Cu and Fe binding sites, protein sequences were submitted to the PHYRE2 protein fold recognition server to identify homologues for known protein structures.^43^ Significant matches (>95% confidence) were submitted to metalPDB^44^ which models the 3D minimal functional sites (MFSs) surrounding metals. Metals were assigned if the search returned significant sequence similarity to metal-binding sites in other proteins (*E*-value <10^−5^). To confirm that genome-predicted proteomes were reflective of expressed proteome metal demands, we performed the same analyses with available published expressed proteomes (Supplementary Figs. 4 and  5). We used both marine and coastal AOA and AOB in our analyses as they were shown to be statistically indistinguishable in terms of genome-predicted proteome metal demand (see Supplementary Fig. 6 and Supplementary Methods for further clarification).

To annotate metal transporter families, we adopted an approach similar to previous studies^45^ where conserved domains were identified using the NCBI-conserved domains database^46^ and were then searched against currently characterised metal transport systems (full list in Supplementary Material). Orthologues were identified using a BLAST search (*E*-value <10^−5^). Each presence of a transporter family gene was considered as a ‘count’ and count numbers were plotted against species. Genomes and transporter families included in this analysis are discussed in greater detail in the Supplementary Material.

### Data analysis

All statistical analyses were performed using Minitab v.13.1. Data were examined for normality and equal variance prior to Student’s *T* test, analysis of variance (ANOVA) or Mann–Whitney *U* statistical tests. Significant results are reported at the 95% confidence level (*P* < 0.05) unless otherwise stated.

## Results and discussion

### Comparison of *N. maritimus* and *N. oceani* response to Fe´ and Cu^2+^

We examined and compared the effect of unchelated Fe´ and Cu^2+^ on the growth of the ammonia-oxidising bacterium, *N. oceani* strain C-107 with that of the archaeal isolate, *N. maritimus* strain SCM1 previously published.^21,22^ *N. oceani* required 0.09 pmol L^−1^ Cu^2+^ to reach maximum specific growth rates (μ_max_) of 0.61 d^−1^ (±0.04), becoming growth-limited at <0.09 pmol L^−1^ Cu^2+^ and ceasing all growth at ≤ 0.05 pmol L^−1^ Cu^2+^ (Fig. 1). At μ_max_, *N. oceani* cells contained 3.1 × 10^−19^ mol Cu cell^−1^ (±1.9 × 10^−19^), equating to a Cu:P of 0.13 mmol mol^−1^. In contrast, *N. maritimus* requires a greater [Cu^2+^] to reach μ_max_ (1 pmol L^−1^ Cu^2+^) but is able to maintain growth at <0.05 pmol L^−1^ Cu^2+^.^21^ At μ_max_, we found that *N. maritimus* had a greater cellular Cu quota of 4.9 × 10^−18^ mol Cu cell^−1^ (±1.9 × 10^−19^) compared with *N. oceani* at μ_max_ (Mann–Whitney *U* test). To our knowledge, *N.* *maritimus* has the greatest cellular Cu:P quota of 1.89 mmol mol^−1^ documented for any marine microorganism growing at μ_max_. Maximum growth rates in *N. oceani* between 0.09 and 0.14 pmol L^−1^ Cu^2+^ may appear to result from the expression of a high-affinity Cu uptake system in a similar mechanism to that demonstrated for Fe.^47^ As *N. oceani* does not possess any known genes for high-affinity Cu uptake (e.g. CTR or *ctaA*—see below for further discussion), we posit that the reduction in μ at >0.14 pmol L^−1^ Cu^2+^ is more likely a toxicity response. Our results suggest that *N. oceani* is more susceptible to Cu^2+^ toxicity than *N. maritimus*: at 53.54 pmol L^−1^ Cu^2+^, *N. oceani* growth was 28% below μ_max_ compared with *N. maritimus* growth rate that was only reduced by 10% at the equivalent [Cu^2+^].

While *N. maritimus* requires an elevated [Cu^2+^] to reach μ_max_ and is able to maintain growth at lower [Cu^2+^] compared with *N. oceani*, we observed the opposite trend between the two microorganisms with respect to Fe´. *N. oceani* reached μ_max_ at 5006 pmol L^−1^ Fe´ and became partially growth limited in treatments with ≤ 828 pmol L^−1^ (Fig. 1). *N. oceani* growth was not detected in treatments with < 100 pmol L^−1^ Fe´. At μ_max_, the intracellular Fe quota of *N. oceani* cells was 6.1 × 10^−17^ mol Fe cell^−1^ (±1.1 × 10^−17^). In contrast, *N. maritimus* reaches μ_max_ at a lower [Fe´] of 550 pmol L^−1^, and is unable to grow at < 250 pmol L^−1^ Fe´.^22^ At μ_max_, *N. maritimus* cells have an intracellular Fe quota of 6.5 × 10^−18^ mol Fe cell^−1^ (±1.3 × 10^−18^)—an order of magnitude less than intracellular Fe quota of *N. oceani* at μ_max_ (Student's *T*-test, two-tailed, *P* < 0.01). The *N. oceani* steady-state Fe´ uptake rate constant (*K*_*in*_), an indicator of Fe affinity, is typical based on the linear relationship between *K*_*in*_ and S.A. in marine phytoplankton and heterotrophic bacteria.^48^ *N. maritimus* deviates from the linear relationship between *K*_*in*_ and S.A. with a lower *K*_*in*_ of 4.5 × 10^−13^ L cell^−1^ h^−1^ (±1.2 × 10^−13^) and therefore per unit S.A. *N. maritimus* has a lower Fe´ uptake rate compared with *N. oceani* and cosmopolitan marine microorganisms (Supplementary Fig. 7).

### Metal requirements and uptake strategies across AOA and AOB communities

**Fig. 2:**
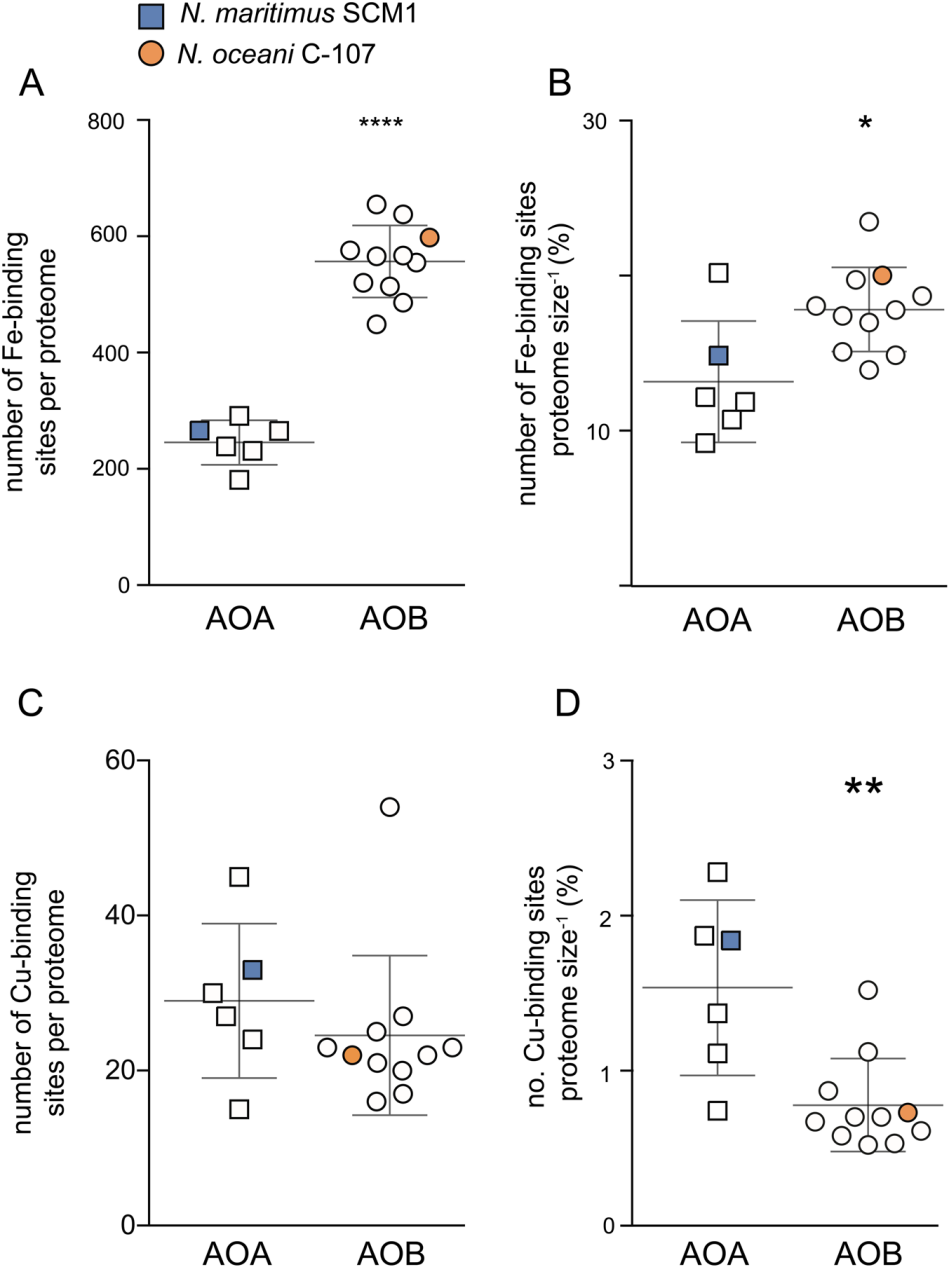
Absolute and relative number of iron- and copper- binding sites in the genome-predicted proteomes of *Nitrosopumilus maritimus* and *Nitrosococcus oceani*. (**A**) Absolute number of Fe-binding sites per genome-predicted proteome of marine (coastal and open ocean) AOA and AOB. See Supplementary Materials for full discussion of included species. **** denotes that values are significantly different between AOA and AOB (Student's *T*-test, two-tailed, *P* < 0.0001). (**B**) Number of Fe-binding sites annotated in the genome-predicted proteome of marine AOA and AOB relative to proteome size (number of proteins predicted from the genome) as a percentage. * denotes that values are significantly different between AOA and AOB (*T* test, two-tailed, *P* < 0.05). (**C**) Absolute number of Cu-binding sites annotated in the genome-predicted proteome of marine (coastal and open ocean) AOA and AOB. See Supplementary Materials for full discussion of included species. (**D**) Number of Cu-binding sites annotated in the genome-predicted proteome of marine AOA and AOB relative to proteome size (number of proteins predicted from the genome) as a percentage. ** denotes that values are significantly different between AOA and AOB (*T* test, two-tailed, *P* < 0.01). For plots **A**–**D**, AOA *n* = 6, AOB *n* = 11. Error bars indicate standard deviation and the middle line indicates mean. All data are available in Supplementary Table 1.

**Fig. 3:**
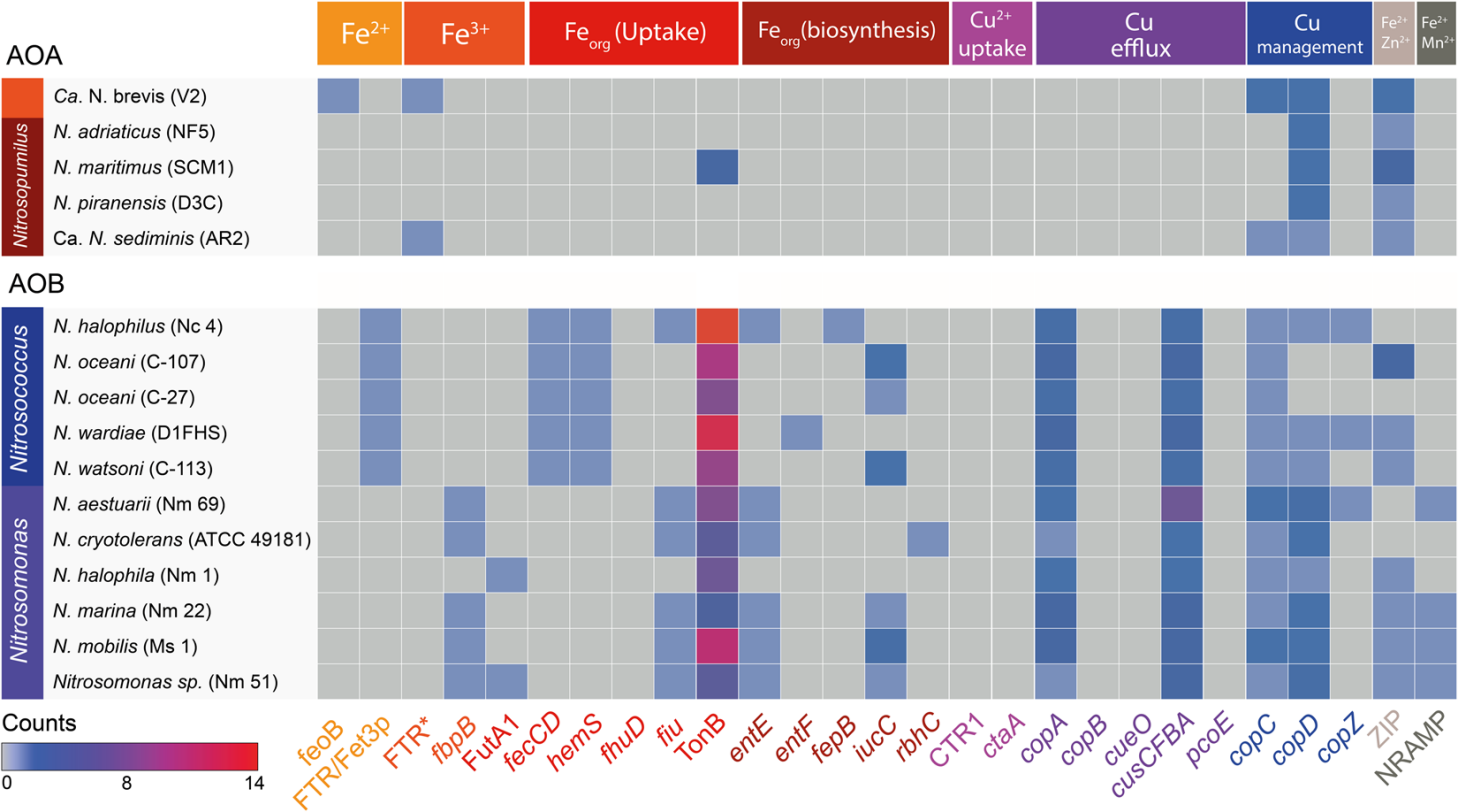
**Presence of genes for metal uptake systems, siderophore uptake and biosynthesis systems, and copper toxicity management systems in marine AOA and AOB genomes, with colours responding to the number of counts**. Metal uptake systems are partitioned by colour based on their metal substrate. A full description of metal transport systems searched in the analysis and data are outlined in Supplementary Table 1. AOA and AOB are partitioned by genus. Strains are given in brackets alongside species names. *FTR refers to the presence of FTR1 gene only without the corresponding fet3p gene that constitutes high-affinity Fe^3+^ uptake.

To explore whether Cu and Fe quotas in *N. oceani* and *N. maritimus* at μ_max_ (Fig. 1) are likely to be representative of broader AOA and AOB communities, we analysed the number of Cu- and Fe-binding sites in available marine AOA and AOB genome-predicted proteomes. We found that the number of hypothetical Fe-binding sites in genome-predicted proteomes were significantly greater in marine AOB compared to AOA (Fig. 2, Student’s *T-*test, *P* < 0.0001, Fig. 3). As such, it is plausible that the greater measured Fe quota at μ_max_ in *N. oceani* is reflective of broader marine AOA and AOB communities, supporting the notion that a greater utilisation of cytochromes^19,20^ burdens AOB with a significant additional Fe demand relative to AOA. The same trend is evident when normalised to proteome size (*P* = 0.006), indicating that the greater number of Fe-binding sites is not an artefact of the larger average genome size in AOB. We did not observe a significant difference between the absolute number of Cu- binding sites in genome-predicted proteomes of AOA and AOB (Fig. 2). Individually *N. oceani* and *N. maritimus* differ in the number of Cu-binding sites per genome—33 in *N. maritimus* versus only 22 in *N. oceani*. Therefore, we speculate that the greater Cu quota per cell in *N. maritimus* compared with *N. oceani* reflects species-level differences and does not necessarily represent trends among the wider AOA and AOB community. However, when normalised to proteome size, AOA are enriched in Cu-binding proteins, suggesting that greater Cu:P in *N. maritimus* compared with *N. oceani* (Fig. 1) may be reflective of broader AOA and AOB communities. A greater Cu:P in *N. maritimus* compared to AOB supports genome and proteome analysis revealing relative to their small proteome/genome size, AOA have an unusually high number of Cu proteins, including plastocyanins and multi-copper oxidases with a putative role in electron transport.^18,49^

### Inorganic Fe uptake

The Fe´ uptake rate constant normalised to S.A. in *N. oceani* exceeds numerous marine microorganisms including *N. maritimus* (Supplementary Fig. 7), suggesting that if illustrative of wider AOB physiologies, AOB may be effective competitors for Fe´ in the marine environment. Analyses of the trace metal uptake complement of available AOB genomes support the case for *N. oceani* C-107 as a suitable representative for wider AOB physiologies: all AOB in our analysis had at least one inorganic Fe-specific uptake system, compared with only 40% of AOA (Fig. 3 and Supplementary Fig. 8). The genomes of all *Nitrosococcus* species encode proteins for the high-affinity FTR/Fet3p Fe^2+^ uptake system (Fig. 3), comprising a multicopper oxidase (Fet3p) that oxidises Fe^2+^ to Fe^3+^ prior to uptake across the inner membrane by FTR.^48^ Given that the main Fe´ substrate in EDTA-buffered systems is Fe^3+^, we predict that Fe^3+^ is reduced prior to being delivered to the FTR/Fet3p system for re-oxidation as demonstrated in previous work.^50^ FTR has high substrate specificity for Fe^3+^ which has been oxidised by the Fet3p multicopper oxidase and as such exogenous Fe^3+^ cannot be provided directly to FTR for uptake.^50^ As we did not observe any reduction in growth rate with the addition of ferrozine (FZ) (Supplementary Fig. 9), any Fe^3+^ reduction does likely not occur extracellularly but rather in the periplasmic space before uptake.

In contrast, *N. maritimus* does not possess any genes diagnostic of known inorganic Fe-uptake systems suggesting a reliance on general cation ZIP transporters for Fe^2+^ uptake or the utilisation of a hitherto uncharacterised Fe-specific transporter. A low Fe´ steady-state uptake rate in *N. maritimus* (Fig. 1) points to Fe uptake by non-specific ZIP transporters rather than an uncharacterised Fe-specific transporter. *N. maritimus* SCM1 was isolated from a marine aquarium,^33^ calling into question whether the Fe uptake strategies seen in *N. maritimus* are reflective of the environmental AOA populations or whether species such as *Ca*. *Nitrosopelagicus brevis*,^49^ with Fe-specific uptake transporters (Fig. 3) are in fact more typical of oceanic AOA. Previous work shows that *N. maritimus* is closely related to marine AOA (in situ and those recovered in enrichment cultures) based on gene content and sequence^7,8,18,51,52^ and meta-genomic and transcriptomic analyses point to the dominance of *Thaumarchaeota* belonging to ‘*Nitrosopumilus*-type’ *Nitrospumilales* in the oceans.^53–55^ In addition, Thaumarcheotal genes homologous to non-specific metal transporters in *N. maritimus* have been shown to be more prevalent in the TARA metagenomic database compared with Ca. *N. brevis*-related Fe transporters.^22^ Together these results suggest that *N. maritimus* may be more representative of broader marine AOA Fe uptake dynamics; however, it is still plausible that Fe uptake strategies play into the niche separation of AOA ‘ecotypes’ or clades in a manner similar to NH_4_^+^, light and oxygen concentrations^56–59^ and provide interesting avenues for future research.

### Chelated iron uptake and siderophore biosynthesis

Despite that fact that AOB isolates appear to be more competitive for Fe´ compared with AOA isolates, Fe´ is still exceedingly scarce in the marine environments with >99.9% Fe complexed to organic ligands.^60^ *N. maritimus* has been shown to be able to utilise Fe even when strongly chelated to the siderophore desferrioxamine B mesylate (DFB), using an extracellular reductive uptake strategy, but shows no physiological and bioinformatic evidence of producing siderophores endogenously (^22^, Fig. 3). An inability to produce siderophores across the *Thaumarchaeota* phylum is suggested by the absence of known siderophore biosynthesis genes in all AOA genomes we examined (Fig. 3; Supplementary Fig. 8). In contrast, the *N. oceani* genome encodes an aerobactin biosynthesis pathway,^61^ a capability likely widespread across AOB: 88% of marine AOB genomes have at least one gene diagnostic of a siderophore biosynthetic pathway (Fig. 3). We did not observe any growth of *N. oceani* in medium wherein all Fe was chelated to a xenosiderophore, desferrioxamine B (Supplementary Fig. 9). However, all but one of the AOB genomes examined encoded multiple TonB-dependent transporters and at least one periplasmic substrate-binding protein capable of transporting siderophores across the outer and inner bacterial membrane (e.g. *fecCD, fhuD, fiu*—see Supplementary Table for full list). The only AOA genome that encodes TonB-dependent transport is *N. maritimus*—the function of this transporter remains to be investigated given that *N. maritimus* reduces Fe extracellularly.^22^ However, all AOB genomes that we examined (except *N. halophilia* Nm 1) encode multiple TonB-dependent transporters and at least one periplasmic substrate-binding protein capable of transporting siderophores across the outer and inner bacterial membrane, respectively (Fig. 3 and Supplementary Fig. 8). Addition of ferrozine (FZ) in EDTA-buffered cultures did not inhibit *N. oceani* growth rates (Supplementary Fig. 9), suggesting that *N. oceani* does not employ a reductive Fe uptake strategy and we did not find any bioinformatic evidence for extracellular ferrireductases in *N. oceani* or AOB genomes. Taken together, these results suggest that AOB are only able to access specific forms of Fe chelates, utilising a direct uptake strategy rather than reducing Fe^3+^ to Fe^2+^ from Fe chelates extracellularly as suggested for *N. maritimus.*^22^ The ferrireductase responsible for reductive uptake in *N. maritimus* has not yet been identified and other AOA genomes do not encode typical ferrireductases (e.g. FRE or SIP), hampering efforts to explore the extent of a reductive uptake strategy among the wider AOA community. If a non-discriminate reductive uptake strategy is widespread among AOA, it may provide a key competitive advantage over AOB that are constrained to specific Fe chelates and Fe´ to satisfy Fe demand. Although AOB are able to respond to Fe scarcity by producing siderophores, siderophores are subject to diffusive losses in the marine environment^62^ and can be taken up by other ‘cheating’ microorganisms. In the oligotrophic ocean a ‘generalist’ reductive strategy as seen in *N. maritimus*, allowing access to a broader range of Fe substrates, may be a favourable strategy over the siderophore production^63,64^ and direct uptake of distinct Fe complexes of *N. oceani* and other AOB.

### Cu uptake and management

The ability of *N. maritimus* to maintain growth under lower [Cu^2+^] compared with AOB points to the presence of a high-affinity Cu uptake system such as CTR1, yet our analysis did not reveal the presence of such systems in either of the examined AOA or AOB genomes across all environments (Supplementary Table). A higher affinity for Cu^2+^ in *N. maritimus* has been hypothesised to be due to the chemical characteristics of their S-layer: Cu^2+^ is characterised as a ‘soft’ metal and is therefore attracted to polarisable ‘soft’ thiol ligands within the archaeal S-layer, accumulating Cu^2+^ at the cell surface to greater concentrations.^36,65,66^ The distinctive S-layer is common to AOA and absent from AOB across all environments—indicating that an ability to grow under low Cu^2+^ may be widespread among *Thaumarchaeota*. Indeed, the soil archaeon *Nitrososphaera viennensis* and other AOA can maintain growth in [Cu^2+^] as low as 10^−4^ pmol L^−1^ Cu^2+^,^67–69^ nearly two orders of magnitude lower than [Cu^2+^] needed to support *N. maritimus* growth. Previous work shows that the soil AOB, *Nitrosomonas europaea*, can maintain growth at [Cu^2+^] below concentrations that are limiting to soil AOA^67^ signifying that environment-specific selection pressures may be at play and that there is likely species-specific physiological diversity within AOA and AOB groups. For instance, high nutrient environments may be less likely to select for S-layer proteins that are more chemically attractive to cations, contributing to the high diversity observed in S-layer proteins.^66^ Compared with *N. oceani, N. maritimus* is able to tolerate greater [Cu^2+^] before an inhibitory effect on growth is observed (^21^; Fig. 1). Environmental studies mirror these results, showing that elevated Cu in marine sediments, river sediments and soils drives a greater reduction in AOB *amoA* gene abundances compared to AOA *amoA* gene abundances.^70–73^ Elevated intracellular copper leads to the production of reactive oxygen species (ROS) that can cause lipid peroxidation, protein oxidation and DNA damage necessitating the production of oxidative defence mechanisms such as superoxide dismutase or catalase. While both AOA and AOB encode genes for superoxide dismutase, the ability to produce the hydrogen peroxide-detoxifying enzyme catalase is absent from the genomes of AOA,^73^ suggesting this is not the mechanism conferring increased Cu^2+^ resistance to AOA.

Our analysis revealed the presence of at least two known Cu efflux and internal Cu management systems in all AOB. These systems include the Cu-exporting CopA,^74^ and CusA^75^ families and the cytoplasm Cu chaperone CopC and CopD,^76^ and CopZ^77^ families. In contrast, AOA genomes do not encode any Cu efflux proteins, and only contain *copC* and/or *copD* genes that play a putative role in chaperoning Cu within the cell.^76^ The rarity of Cu efflux systems is not unique to *Thaumarchaeota*: genomes across the Archaeal domain encode significantly fewer Cu efflux genes compared to genomes of the Bacterial domain^78^ calling into question the mechanism allowing AOA isolates to tolerate greater [Cu^2+^] in culture (Fig. 1). Amin et al.^21^ found that under Cu-replete conditions, *N. maritimus* produced weak ligands that were not produced under Cu-limiting conditions. The production of ligands with a range of stability constants, which form complexes with free Cu^2+^ thereby preventing it from accumulating on cell surfaces, is a common indirect strategy among marine microorganisms to manage high Cu^2+^.^79–81^ If the ligands produced by *N. maritimus* are playing a similar role in lowering cupric ion activity, it explains how *N. maritimus* is able to tolerate higher (calculated) [Cu^2+^], despite the lack of known Cu-detoxification genes in its genome.

### Implications for marine ammonia oxidation, niche separation and evolution of ammonia oxidation

#### Copper limitation of ammonia oxidation

While ‘omics approaches are useful in making initial predictions about microbial niche separation, the effect of genotypic variation on physiology is more robustly gleaned from examining pure isolates in controlled culture conditions—a non-trivial task given that pure isolates of AOA and AOB are lacking. If representative of in situ communities, as suggested by our bioinformatic analysis of available genomes, our physiological characterisation of *N. oceani* C-107 and *N. maritimus* SCM1 provides testable hypotheses and new avenues to be explored in future research.

**Fig. 4:**
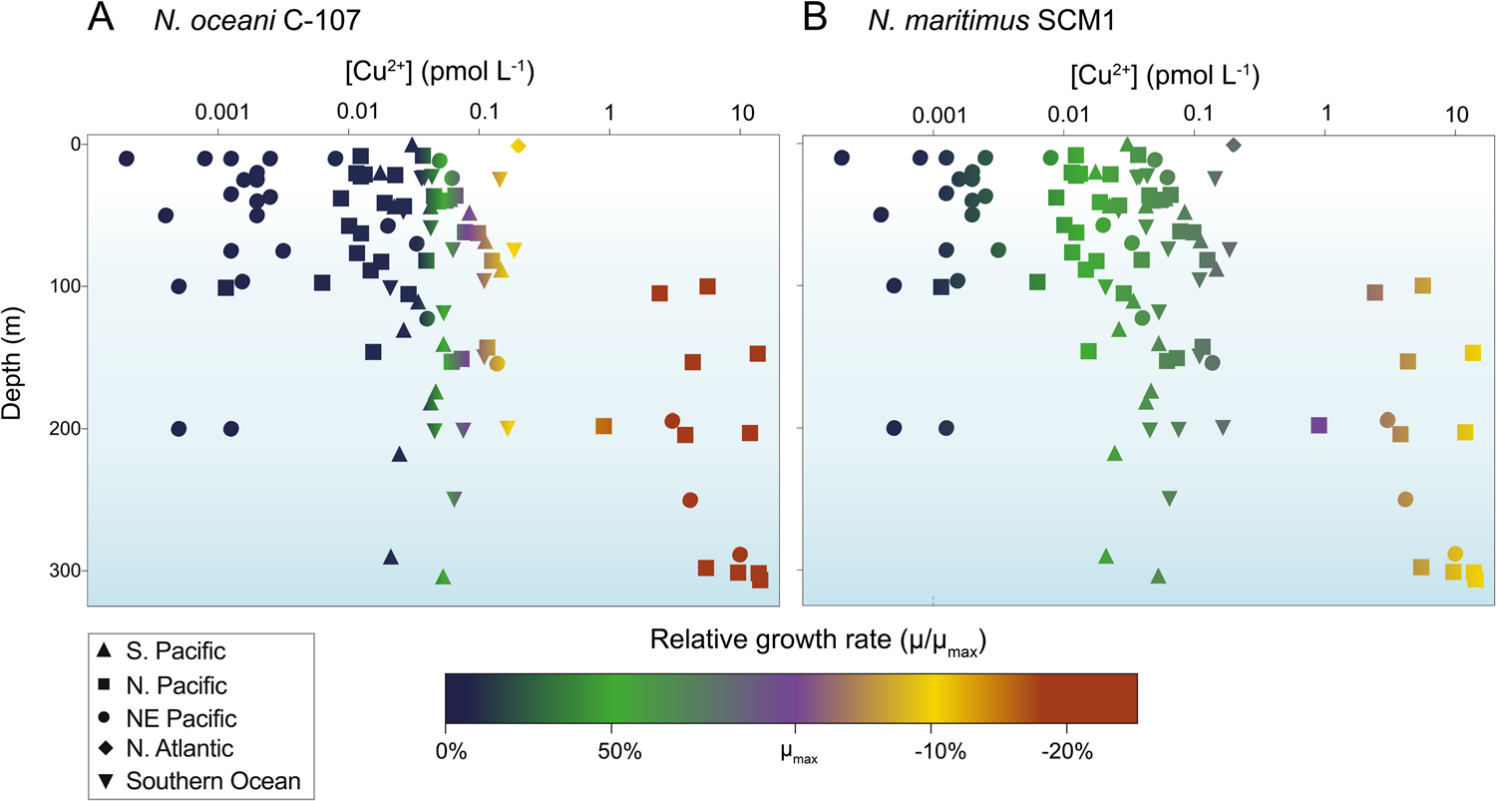
Laboratory-based growth rate of the marine AOA isolate *Nitrosopumilus maritimus* and the AOB isolate *Nitrosococcus oceani* at cupric ion concentrations measured in the marine environment. Published oceanic cupric ion concentrations ([Cu^2+^]) (see Supplementary Table 1 for all references) plotted against a colour gradient reflecting the relative growth rate of (**A**) *N. oceani* C-107 and (**B**) *N. maritimus* SCM1 in culture at the given [Cu^2+^]. For instance, at [Cu^2+^] measured in the North East Pacific (on average between 0.001 and 0.01 pmol L^−1^ Cu^2+^), no growth of *N. oceani* is observed in culture (Fig. 1), hence NE Pacific data points are largely dark blue (no growth). Growth data for *N. oceani* were obtained from this study (Fig. 1), and data for *N. maritimus* were previously published.^21^

To our knowledge the cupric ion concentration ([Cu^2+^]) required for minimum growth in *N. oceani* C-107 and *N. maritimus* SCM1, is greater than all other microbial components of the marine N-cycle,^21,26,82–85^ (Supplementary Table 1 and Supplementary Fig. 10) examined to date, suggesting that ammonia oxidation may act as a bottleneck in marine biogeochemical N-cycling when [Cu^2+^] is low. We hypothesise that ammonia oxidation in the Pacific Ocean may be susceptible to Cu limitation, as [Cu^2+^] measured in the Pacific can be spatially and temporally below the AOA and AOB Cu-limiting threshold (<0.01 pmol L^−1^ Cu^2+^) (Fig. 4). In the North East Pacific, where [Cu^2+^] has been documented to be < 0.001 pmol L^−1^ Cu^2+^ at all depths,^86,87^ Cu limitation may extend throughout the water column, contributing to the low observed ammonia-oxidation rates and *amoA* gene abundances despite otherwise replete NH_4_^+^.^13^ Cu-amendment experiments conducted in situ will be necessary in order to test this hypothesis as open-ocean ecotypes may harbour physiologies distinct to those demonstrated here.

The observation that AOB become completely growth limited at greater [Cu^2+^] relative to the AOA aligns with observations of lower AOB: AOA abundances under Cu-deplete regimes in the North East Pacific (0.0001–0.01 pmol L^−1^ Cu^2+^), despite concentrations of NH_4_^+^ which are otherwise sufficient to support AOB growth.^14^ [Cu^2+^] ranges over five orders of magnitude between the surface and deep ocean **(**Fig. 4) (0.0001–10 pmol L^−1^ Cu^2+^) and varies three orders of magnitude between ocean basins (0.0010 pmol^−1^ Cu^2+^ in the Pacific^86,87^ to 0.2 pmol L^−1^ Cu^2+^ in the North Atlantic^88^). The ability of *N. maritimus* to support growth over a broader [Cu^2+^] range, may provide an additional adaptation, in addition to a higher affinity for NH_4_^+^,^14^ which contributes to the greater success of AOA in the environment.

#### AOA and AOB trace metal niche separation

**Fig. 5:**
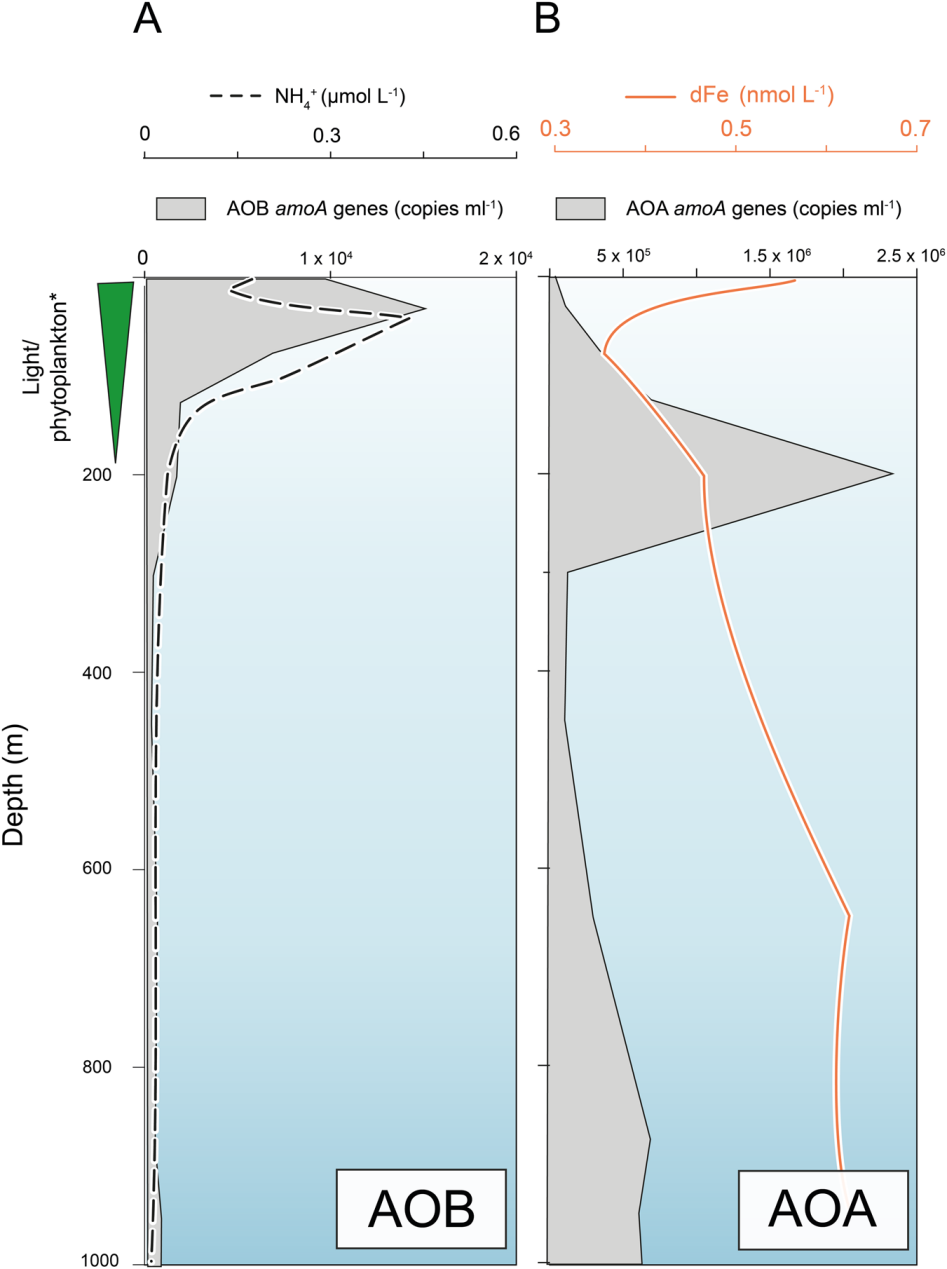
Schematic showing hypothesised spatial niches of AOB and AOA. Published *amoA* gene abundance depth profiles (shaded grey area—copies ml^−1^ references provided in Supplementary Table 1) for AOB (**A**) and AOB (**B**) are plotted against NH_4_^+^ depth profiles (black dashed line—µmol l^−1^ from ref. ^88^) and dissolved Fe, dFe (orange solid line—nmol L^−1^, data from GEOTRACES Intermediate Data Product 2017). Green wedge labelled light/phytoplankton* reflects the relative decrease in light, phytoplankton and marine bacteria with increasing depth.

As AOB have a lower affinity for NH_4_^+^ compared with AOA,^14^ it follows that AOB must occupy a niche with a high NH_4_^+^ supply to fulfil their NH_4_^+^ demand. NH_4_^+^ depth profiles show that NH_4_^+^ begins to accumulate to appreciable levels within the photic zone between 50 and 100 m (Fig. 5), as light limitation of phytoplankton growth causes NH_4_^+ ^consumption to slow.^89,90^ Compiled depth profiles show that AOB *amoA* abundances also peak below 100m depth (Fig. 5 and Supplementary Fig. 11), shallower than AOA, pointing to the greater role of NH_4_^+^ supply in governing the vertical distribution of AOB. A need to be proximal to high NH_4_^+^ supply in shallower waters would force AOB to conform to the trace metal environment of sunlit surface waters: scarce Fe with fierce competition with phytoplankton and heterotrophic bacteria. A high affinity for Fe´ demonstrated in *N. oceani*, and the presence of Fe-specific transporters among the wider AOB community (Fig. 3), suggests that AOB would be effective competitors with marine phytoplankton and bacteria for unchelated Fe in the photic zone. AOB would also be able to respond to chronic Fe scarcity in upper surface waters by producing their own siderophores to enhance access to Fe. A niche for AOB in the sunlit upper water column is also supported by evidence indicating that AOB have a higher tolerance to irradiance than AOA.^3,91^ At shallower depths, AOB would avoid any toxic effects of Cu^2+^ deeper in the water column: below 200m depth[Cu^2+^] (>1 pmol L^−1^ Cu^2+^) is sufficient to cause partial inhibition of *N. oceani* growth in culture (Fig. 4). The potential for Cu toxicity is supported by observations of environmental genes and transcripts—homologous to AOB-Cu efflux proteins—which show a general scaling between Cu^2+^ concentration (shown in Fig. 4) and read abundance (Supplementary Figs. 12 and  13). For example, highest reads (as a percentage of total reads) are observed in the North Atlantic and North Pacific where Cu^2+^ can reach 0.2 pmol L^−1^ Cu^2+^.

In addition to explaining why AOA widely outnumber AOB, a higher affinity for NH_4_^+^ compared with AOB^14^ affords AOA the luxury of occupying greater depths wherein the NH_4_^+^ supply is lower, but photoinhibitory effects are substantially reduced. Indeed, AOA maxima are commonly at the base of the photic zone^8,11,92^ deeper than AOB maxima (Fig. 5 and Supplementary Fig. 11). With increasing depth, competition for Fe would lessen as phytoplankton, heterotrophic bacteria and AOB become limited by light, DOC^93^ or NH_4_^+^, respectively. As such, below the photic zone, there would be little selective pressure to develop a high-affinity Fe´ uptake system—reflected in the rarity of inorganic Fe´ transporters among marine AOA and the low steady-state uptake rate of Fe´ in *N. maritimus*. As well as the reduction in competition for Fe, with increasing depth, the water column becomes naturally richer in dissolved Fe as organic matter remineralises (Fig. 5). The capacity to reduce Fe chelates via a reductive strategy^22^ would make AOA well adapted to utilise organic Fe chelates from remineralising organic matter, which are heterogeneous in nature.^60^ An ecological niche driven by Fe remineralisation, which is deeper than N remineralisation, potentially explains why AOA abundances and nitrification rates correlate with depth-integrated primary productivity but do not respond to additions of NH_4_^+^ in open-ocean environments.^11,13^

#### Geological implications

Molecular clock analyses constrain the timing of marine AOA and AOB emergence to 1084–959 million years ago (Mya) and 1169–596 Mya,^94,95^ respectively. The overlapping error of such approaches demands additional tools to constrain the emergence of archaeal and bacterial ammonia oxidisers. Consideration of evolving trace metal availabilities in the ocean over geologic time offers an approach that may complement molecular clock analyses and provide new insight into the relative timing of the emergence of AOA and AOB. The progressive oxygenation of the oceans over geologic time has driven changes in the bioavailability of essential trace metals, notably enhancing the availability of Cu and Zn due to metal sulphide dissolution but drastically reducing the availability of Fe, Co and Mn that are insoluble under oxic conditions.^27,28^ The result is a shift in microbial metal usage as organisms evolve to utilise more accessible metals, leaving imprints in the genomes, proteomes and metallomes of extant microorganisms.^27–30,96^ Considered in this context, a greater Fe demand and higher sensitivity to Cu points to an earlier emergence of the AOB and additionally supports a major transition in trace metal availability between 1084 and 596 Mya driven by oxygenation^28^ to impart such differences in AOA and AOB trace metal composition. Although AOA extensively outnumber AOB in the modern open oceans, the greater availability of Fe may have allowed AOB to play a more prominent role in the Precambrian aerobic nitrogen cycle. The role of oxygenation in liberating bio-essential trace metals such as Cu and Mo, thereby lifting limitation of certain metabolic pathways and allowing microorganisms to flourish into new trace metal niches, has previously been suggested for N_2_ fixation,^97^ but may have also influenced nitrification. A drawdown in bioavailable Fe and increase in Cu availability with oxygenation would allow AOA to proliferate into the newly available chemical niche thereby becoming the key players in the aerobic N-cycle, owing to their lower Fe cellular quota and greater tolerance of Cu. At this point, the contribution of AOB may have been reduced due to their greater Fe demand, now scarce in a widely oxygenated ocean.

## Conclusions

The results presented here provide the first parallel physiological and bioinformatic comparison of marine AOA and AOB Fe and Cu requirements. We hypothesise that a greater affinity for unchelated Fe allows AOB to occupy shallower waters to fulfil their high NH_4_^+^ demand, while the strategy of reducing Fe chelates would make AOA better suited to below the photic zone where Fe supply from remineralisation is greater. Further, we posit that the ability of AOA to tolerate a broad range of Cu concentrations compared with AOB contributes to the global success of AOA as Cu concentrations are highly variable in the marine environment. Considered in the context of evolving trace metal availability over geologic time, our results offer a new perspective to an ongoing debate—suggesting that the greater Fe demand but lower Cu tolerance of AOB compared with AOA is vestigial to the dominance of AOB in the Precambrian when the Fe concentrations were greater but Cu availability was lower.

## Supplementary information

Supplementary information

Supplementary table 1
