# Supplementary material for: Marine ammonia-oxidising archaea and bacteria occupy distinct iron and copper niches
Source: ISME Commun. 2021 Mar 24;1:1. doi: 10.1038/s43705-021-00001-7 (PMC9723733; doi:10.1038/s43705-021-00001-7)
Supplement: Supplementary file 1 — Supplementary information [file 43705_2021_1_MOESM1_ESM.docx]

**Supplementary Materials for**

**Marine ammonia-oxidising archaea and bacteria occupy distinct iron and copper niches**

Roxana T. Shafiee*^1^, Poppy J. Diver^1^, Joseph T. Snow^1^, Qiong Zhang^1^, Rosalind E.M. Rickaby^1^

^1^ Department of Earth Sciences, South Parks Road, University of Oxford, Oxfordshire, OX1 3AN, United Kingdom.

***Corresponding author details**

Roxana T. Shafiee

Department of Earth Sciences, South Parks Road, University of Oxford, Oxfordshire, OX1 3AN, United Kingdom

[roxana.shafiee@earth.ox.ac.uk](mailto:roxana.shafiee@earth.ox.ac.uk)

**Supplementary Methods**

***SCM1 cultures.*** Polycarbonate culture vessels and culturing apparatus were acid cleaned in 10% (v/v) TraceMetal™ grade HCl (Fisher Scientific, Loughborough, UK) for 24 hours and UV-sterilised before use. Triplicate cultures of *Nitrosopumilus maritimus* strain SCM1 were maintained as previously described (Amin *et al*, 2013; Shafiee *et al*, 2019) for the purpose of intracellular Cu-quota analysis. Basal salt medium and macronutrients were treated with Chelex-100 resin (BioRad, Watford, UK) to remove trace metal contaminants (Price *et al*, 1989) and passed through an acid-washed (10% HCl v/v) 0.1 µm polycarbonate-filter to sterilise in a metal-free clean laboratory. The final pH of the media was 7.5. Trace metals were added from filter-sterilised concentrated stocks made using >99.999% trace metal basis salts (Sigma Aldrich, UK) and maintaining a constant concentration of at 12 µmol L^-1^  EDTA. Previous studies have not found an inhibitory effect on *N. maritimus* growth with the addition of 12 μmol L^−1^ EDTA, relative to regular SCM media with 7.5 μmol L^-1^  EDTA. Fe´, Cu´ and cupric ion (Cu^2+^) concentrations were calculated using Visual Minteq software (Gustafsson, 2010). Background Fe and Cu contamination of 7.3 and 12.5 nmol L-1, respectively, from basal SCM medium was measured using inductively coupled plasma mass spectrometry (Zhang *et al*, 2018). We were interested in determining the *N. maritimus* Cu quota at the first instance that maximum growth (μ_max_) is achieved in order to gain an indication of true metal requirement that is not influenced by internal metal storage or growth limitation. As such we acclimated *N. maritimus* to optimal Cu^2+^ concentrations determined using previous work (Amin *et al*, 2013) which showed that *N. maritimus* reaches μ_max_ at 1pM Cu^2+^. Cultures were acclimated to 1pM Cu^2+^ by consecutive transfer during late exponential growth phase into new media until growth rates did not vary with statistical significance (ANOVA, p<0.01). Specific growth rate (d^-1^) was calculated over the linear phase of semi-log plots of nitrite concentration over time, determined spectrophotometrically (Griess, 1879). Axenicity was tested by SEM imaging of cultures. *N. maritimus* cultures acclimated to 1pM Cu^2+^ were used to inoculate larger 2 L triplicate cultures for trace metal quota analysis. Cultures were harvested during mid-exponential phase for trace metal analysis as per discussion below.

***Trace metal quotas.*** Intracellular metal quotas were determined at the [Fe´] and [Cu^2+^] that maximum growth, μ_max_, is first achieved in order ensure that the metal quotas are not elevated through storage under replete conditions but are not depleted due to limitation. [Fe´] and [Cu^2+^] for *N. oceani* C-107 cultures to be harvested for trace metal analysis were therefore informed by initial dose-response curves with 5006 pmol L^-1^  Fe´ and 0.09 pmol L^-1^  Cu^2+^ and 0.13 pmol L^-1^  Cu´ treatments deemed to represent μ_maz_. We also maintained additional cultures of *N. oceani* at 828, 376 and 100 pmol L^-1^  Fe´ and 24.63 pmol L^-1^  Cu^2+^ for trace metal analysis. *N. maritimus* cultures were maintained at 1 pmol L^-1^ Cu^2+^ deemed to be optimal from previous work (Amin *et al*, 2013). All cultures were maintained in triplicate. We were unable to obtain enough biomass to perform trace metal analysis of *N. oceani* and *N. maritimus* Cu-limited cells. Due to the precipitation of Fe hydroxides from the medium, we were not able perform an experiment with high enough Fe´ to obtain metal quotas reflecting Fe –storage in *N. oceani.* For ease of comparison with other marine microorganisms, in Figure 1 of the main manuscript we present the minimum Cu and Fe quotas at μ_max_ normalised to cellular P. Quotas of Cu, Fe and P per cell at varying Fe´ and Cu^2+^ are shown in Supplementary Figure 1 and Supplementary Table.

***SEM imaging.*** Fe-limited (100 pmol L^-1^ Fe´) *N. oceani* C-107 cell cultures were harvested by centrifugation into a concentrated cell suspension were applied to poly-L-lysine coated glass coverslips which were then fixed with 2.5% glutaraldehyde and allowed to adhere for 5 min. Glass coverslips were rinsed 3 x with 0.1M phosphate buffer (pH 7.2) and then underwent secondary fixation with 1% OsO_4_ for 45 min. Cells on coverslips were rinsed with water, dehydrated through a graded ethanol series to absolute ethanol, and then dried chemically with HDMS. Coverslips were mounted on carbon adhesive tape and splutter coated with 10 nm Au. Cells were examined using a GeminiSEM at an accelerating voltage of 3 kV and magnifications of × 5,000 – 100,000. Cell sizes were calculated using imageJ as described previously (Shafiee *et al*, 2019) and are consistent with transmission electron micrographs obtained previously (Klotz *et al* 2006).

***FeDFB and ferrozine experiments.*** To examine whether *N. oceani* can utilise organically chelated Fe, *N. oceani* was cultured in medium with the addition of siderodesferrioxamine B mesylate (DFB; Merck), a fungal siderophore that has a greater stability constant (logK´_FeL, Fe´_ = 11.8) for Fe(III) compared with EDTA (logK´_FeL, Fe´_ = 8.6, pH 8) (Maldonado *et al*, 2005). *N. oceani* was cultured under replete total dissolved Fe, [dFe], (5006 pmol L^-1^  dFe) and low [dFe] (100 pmol L^-1^  dFe). A 1.3-fold excess of DFB was added over Fe, in order to reduce Fe´ to negligible concentrations (<1 x 10^9^ pmol L^-1^  Fe´) thereby making Fe bound to DFB the main Fe substrate. Fe´ in DFB buffered media was calculated using the equation [Fe´] = [FeDFB]/([DFB´] x [K_Fe´DFB_^cond^]) (Maldonado & Price, 2001). EDTA concentrations were kept constant at 12 µmol L^-1^ EDTA to buffer other trace metals. We used the ferrozine assay (Kranzler *et al*, 2011) to identify whether *N. oceani* adopts a reductive Fe uptake pathway in Fe acquisition. *N. oceani* cultures acclimated to replete and deplete Fe´ ( 5006 pmol L^-1^  Fe´ and 100 pmol L^-1^  Fe´) and then were treated with 200 µmol L^-1^ ferrozine (FZ; 5,6-Diphenyl-3-(2-pyridyl)-1,2,4-triazine) at the beginning of experiments. As Fe(II)-FZ_3_ complexes are unavailable for uptake across the plasma membrane, a reduction in growth rate suggests a reductive uptake pathway is present in Fe acquisition. All Fe uptake experiments were performed in the dark to prevent the photoreduction of Fe(III) chelates.

***Metal uptake transporters.*** AOA and AOB sequences from a wide range of environments were downloaded from UniProt and conserved domains were identified using NCBI conserved domains database (Marchler-Bauer *et al*, 2010). Only genome-encoded proteomes with a high genome completeness (as per UniProt BUSCO and CPD analysis) were used in the analysis. Orthologs of conserved metal transport domains (see supplementary table for full list) were BLAST searched against the AOA and AOB (e value < 10^-5^). Conserved domain annotations for the search were identified from reviewing published literature of metal uptake genes and by using the study of Hogle *et al* (2016) as a framework. Our search focussed on genes involved in both periplasmic and inner membrane inorganic Fe (II) and (III) uptake, Fe –complex (siderophore) uptake, Fe –siderophore biosynthesis, Cu-uptake, Cu-internal management and P-type ATPase Cu-efflux. All metal uptake systems and references are outlined in the Supplementary Table. We acknowledge that there may be as of yet unidentified systems which are unique to AOB and AOB which are revealed through deeper future physiological and bioinformatic characterisation of existing and novel AOA/AOB isolates. Due to the low number of available AOA and AOB marine genomes both coastal and open ocean marine species were used in comparative analysis. Although we present data of the trace metal uptake complement of broader AOA and AOB species across all environments in Supplementary Figure 8, its discussion is beyond the scope of this study but we hope that is may be a useful research for future work exploring the trace metal niche separation of AOA and AOB in different environments.

***Annotation of metal binding sites in the genome-predicted proteome.*** The limited number of marine AOA and AOB in pure culture precludes a robust analysis of the trace metal requirements across the wider AOA/AOB community using culture-based approaches. To address this limitation, we adopted a bioinformatic approach to determine whether the intracellular metal quotas measured at μ_max_ in *N. oceani* C-107 and *N. maritimus* SCM1 are representative of the phylogenetically diverse AOA and AOB. We annotated the hypothetical Cu- and Fe- binding sites in genome-predicted proteomes of marine AOA and AOB with the underlying assumption that a greater number of metal binding sites will impart a greater intracellular quota at μ_max_. Genome-predicted proteomes were only used in the analysis with a high genome completeness (>96%). As full genomes of open ocean AOA and AOB are limited and in order to increase our sample size we tested whether number of metal-binding sites in genomes of coastal marine AOA and AOB vary with statistical significance. In AOB, we found that the values did not vary with statistical significance between coastal and open species for either the number of Fe –binding sites (Supplementary Figure 6; unpaired T-test, two-tailed, p = 0.95) the relative number of Fe –binding sites per proteome (unpaired T-test, two tailed, p = 0.4) the number of Cu-binding sites per proteome (unpaired T-test, two tailed, p = 0.41) or the number of Cu- binding sites relative to proteome size (unpaired T-test, two tailed, p = 0.24). As there is only one genome sequence for an open ocean *Thaumarchaeon* (Ca. *N. brevis*) we included genomes of coastal *Thaumarchaeon* in our analysis too – making the assumption that as shown in AOB, the mean number of Fe and Cu binding sites would not vary with statistical significance and that by including coastal AOB in our analysis too, and that any differences arising from the difference in habitat between coastal and open ocean AOA would also influence on metal quotas in AOB. Values were also normalised to total number of proteins per proteome to determine the proportion of the proteome dedicated to Cu or Fe use. Statistical significance in number of metal binding sites between AOA and AOB was examined using a Student’s T-test for parametric datasets (two tailed, p < 0.01) and Mann Whitney U for non-parametric datasets.

In order to confirm that the genome-expressed proteomic metal demand scales expressed proteomic metal demand, we performed the same analysis as described above, using limited available expressed AOA and AOB proteomes (AOB – Zorz *et al* (2018); Pellitteri-Hahn *et al* (2011); Jiang *et al* (2015); AOA – Santoro *et al* (2015); Qin *et al* (2018); Shafiee *et al* (unpublished). To account for the varying methods by which protein abundances are calculated between studies, metal demands were calculated relative to total protein abundance ((number of Fe/Cu-binding sites per protein x protein abundance)/total protein abundance). We found that expressed-proteome Cu and Fe demand is positively correlated with absolute predicted-proteome metal demand (Fe, r^2^ = 0.56, p<0.01; Cu, r^2^ = 0.42, p = 0.06) and therefore the differences between AOA and AOB Fe/Cu demands based on predicted-proteomes as shown in Figure 3, hold true when compared based expressed-proteomes (Supplementary Figure 5).

***Presence of copper efflux genes in the TARA global metagenomes and metatranscriptomes.*** In order to examine for the potential for *in situ* AOB communities to be inhibited by high Cu^2+^ as suggested by our physiological results we screened the Ocean Data Atlas, which mines data from the Tara Oceans metagenomes and metatranscriptomes, for reads of known AOB (including *Nitrosococcus*, *Nitrosomonas* and *Nitrosospira*) Cu efflux proteins – namely *copA* and *copB* (See Supplementary Table for all genes used in the search). As AOA do not utilise Cu efflux proteins, it is not possible to perform a similar search in AOA. The significance level (E) was set as 1e^-10^ and reads were returned as a percentage of total reads. Only reads ascribed to different AOB – either *Nitrosococcus*, *Nitrosospira* and *Nitrosomonas* were considered in subsequent analysis. The search return numerous significant hits of AOB-ascribed reads homologous to AOB-copper efflux proteins– showing these genes are widespread in the world’s oceans (Supplementary Figure 12). The TARA metatranscriptomic data is more limited than the metagenomic data, but many of the returned *Nitrosococcus* and *Nitrosomonas* hits are from metatranscriptomic samples (Supplementary Figure 13) indicating that Cu efflux proteins are actively being expressed in the oceans. The fact that the search returned significant hits ascribed to the *Nitrosococcus*-genera is also support for the ubiquity of *Nitrosococcus* in the world’s oceans and supports its use as a representative species in our study.

***AOA and AOB niche separation.*** In order to place our results in the context of AOA and AOB niche separation, we explored the co-variance of key environmental parameters with AOA and AOB abundance profiles. Figure 5 in the main manuscript shows a schematic of the averaged published AOA and AOB *amoA* gene profiles (Full data available in Supplementary Table and Supplementary Figure 11), dissolved total Fe (dFe) from GEOTRACES Intermediate Data Product 2017 (full data in Supplementary Table) and NH_4_^+^ depth profiles (Gruber, 2008).

***amoA depth profiles.*** The presence of *amoA* genes in DNA extracted from environmental samples gives an indication of the organisms potentially capable of ammonia oxidation, while the presence of *amoA* transcripts indicates the presence of actively ammonia-oxidizing microorganisms. Due to the wealth of AOA and AOB *amoA* gene distribution data, we were able to determine a robust pattern of abundance profiles (Supplementary Figure 11). The fewer number of studies examining *amoA* transcripts — and the complete absence of AOB amoA transcript profiles to our knowledge — precluded us from calculating a complementary *amoA* transcript depth profile. However, the few studies which have simultaneously examined gene copies and transcripts show that peak abundances/expression co-occur (Church *et al*, 2010; Jing *et al*, 2017). Further, studies measuring instantaneous AOA/AOB gene copies and transcripts (rather than depth profiles) typically co-vary such that when more cells are present the number of actively transcribing cells can be assumed to be greater (Abell *et al*, 2011; Tolar *et al*, 2016; Duff *et al*, 2017; Leininger *et al*, 2005) although the ratio of gene copies to transcript may vary depending on the environment. Based on the aforementioned discussion, we are confident that the *amoA* gene profiles provide an accurate picture of the abundance of microorganisms actively oxidising ammonia-oxidising microorganisms.

**Supplementary Results and Discussion**

***AOB Fe´ uptake proteins.*** In contrast to *Nitrosococcus* genomes which all encode the high affinity FTR/Fet3p uptake system, only two genomes of all the *Nitrosomonas* and *Nitrosospira* clade we examined contained FTR/Fet3p uptake genes none of which were marine species (Supplementary Figure 8). We only found periplasmic Fbp or FutA1 Fe^3+^ transporters in marine *Nitrosomonas* and *Nitrosospira*. The FTR1/Fet3p used by *Nitrosococcus* and Fbp /FutA1 used by *Nitrosomonas* and *Nitrospira* differ in their Fe –substrate, using Fe^2+^ and Fe^3+^ respectively. Of the small proportion of marine Fe which is unchelated, Fe primarily exists as Fe^3+^. We hypothesise that the ability of *Nitrosomonas* and *Nitrospira* to take up exogenous Fe^3+^ may provide them a competitive advantage relative to *Nitrosococcus* in terms of inorganic Fe acquisition. Yet Fe^2+^ accumulates to appreciable concentrations in some regions (Roy *et al*, 2008) and as such competition between *Nitrosococcus* and *Nitrosomonas* for unchelated Fe is likely to be spatially and temporally variable. As there are no marine *Nitrosomonas* pure isolates we are not able to demonstrate this differentiation physiologically and therefore this will be an important avenue for future research as cultures become available. Nonetheless, the fact that all examined marine AOB all have Fe-specific (Fe´) uptake mechanisms which we posit confers them with a higher affinity for unchelated Fe´ in the oceans relative to the AOA – none of which have any specific Fe´ uptake transporters and must therefore rely on generalist divalent cation transporters.

**Supplementary Figure 1.**  *N. oceani* C-107 relative growth rate as a function of A) increasing Cu^2^**^+^** (equivalent Cu´ is also shown for comparison) B) increasing Fe´ Cellular Cu (C) and Fe (D) quotas in mol cell^-1^  at different [Cu^2+^] and [Fe´]. Mol Fe cell^-1^  in *N. maritimus* SCM1 is from previously published dataset (Shafiee *et al*, 2019). The [Fe´] and [Cu^2+^] maximum relative growth rate is reached (μ_max_) is denoted by a dashed line – metal quotas under these [Fe´] and [Cu^2+^] are those shown in the main text (Figure 1C). Error bars denote standard deviation. n = 20 in A and B, n = 3 in C and D

**Supplementary Figure 2.** Effect of cupric ion (Cu^2+^) concentration (and corresponding unchelated Cu, Cu´) (A) and unchelated Fe (Fe´) concentration (B)

**Supplementary Figure 3.** Scanning Electron Micrograph (SEM) images of *N. oceani* C-107 under Fe –limited conditions.

**Supplementary Figure 4.** Relationship between metal (Fe – left, Cu – right) demand of calculated from genome-predicted proteome and expressed proteome.

**Supplementary Figure 5.** Comparison of Fe and Cu demand of AOA and AOB (calculated based on expressed-proteome)

**Supplementary Figure 6.** Comparison of the absolute (A) and relative (to proteome size) number of hypothetical Fe –binding sites (B), absolute (C) and relative (to proteome size) number of Cu-binding sites in genome-predicted proteomes of open ocean ammonia oxidising bacteria (blue squares) and coastal ammonia-oxidising bacteria (red circles). Middle line represents the mean value, error bars represent standard deviation. Species-specific values are given in Supplementary Table.

**Supplementary Figure 7.** log Fe´ steady-state uptake rate constant (K_in_) normalised to cellular surface area (S.A.) in *N. oceani* C-107 compared with previously published data of marine phytoplankton and *N. maritimus* SCM1 (Shafiee *et al*, 2019), with greater values indicating a higher affinity for Fe´. Phytoplankton data compiled in Lis *et al* (2015)

**Supplementary Figure 8.** Heatmap of extended bioinformatic analysis of transporter families in AOA and AOB genomes. Colours describe the number of ‘counts’ or occurrences per genome of the given genome/superfamily. Description of uptake systems and further information on the genomes given in Supplementary Table.

**Supplementary Figure 9.** *N. oceani* specific growth rates (d^-1^ ) in 1) EDTA-buffered medium with Fe´ ([Fe´] below bars) as the main Fe –substrate (left panel) 2) desferrioxamine buffered medium with Fe –DFB (organically complexed Fe) as the main Fe –substrate ([FeDBF]. n.g. indicates no growth observed (middle panel). 3) EDTA-buffered medium with Fe´ ([Fe´] below bars) supplemented with Fe(II)-binding ferrozine (FZ) at the beginning of experiments (right panel). Error bars denote standard deviation, n = 6.

**Supplementary Figure 10.** Effect of cupric Cu concentration (Cu^2+^) on relative growth rate (µ/µ_max_) on AOB *N. oceani* C-107 compared with published data on AOA *N. maritimus* strain SCM1 and other marine microorganisms mediating the components of the nitrogen (N)-cycle, with numbers corresponding to pathways shown on the bottom figure of the marine N-cycle. All data available in Supplementary Table.

**Supplementary Figure 11.** Mean AOB and AOA *amoA* gene abundances from data compiled datasets – see Supplementary Table for references.

**Supplementary Figure 12.** AOB-ascribed gene reads homologous to known AOB Cu detoxification genes (See Supplementary Table 1) as a percentage of total reads.

**Supplementary Figure 13.** AOB-ascribed transcript reads homologous to known AOB Cu detoxification genes (See Supplementary Table 1) as a percentage of total reads.

**References**

Abell GC, et al*.* Effects of estuarine sediment hypoxia on nitrogen fluxes and ammonia oxidizer gene transcription. FEMS microbiology ecology.2011: 75: 111-122.

Amin SA, et al. Copper requirements of the ammonia‐oxidizing archaeon Nitrosopumilus maritimus SCM1 and implications for nitrification in the marine environment. Limnol Oceanogr. 2013;58:2037-45.

Brand LE, Sunda WG, Guillard RR. Reduction of marine phytoplankton reproduction rates by copper and cadmium. J Exp Mar Biol Ecol. 1986;96:225-50.

Church MJ, et al. Abundances of crenarchaeal amoA genes and transcripts in the Pacific Ocean. Environ Microbiol. 2010;12:679–88.

Duff AM, Zhang LM, & Smith CJ. Small-scale variation of ammonia oxidisers within intertidal sediments dominated by ammonia-oxidising bacteria Nitrosomonas sp. amoA genes and transcripts. Sci Rep.2017; 20: 2834-2853.

Dupont CL, Yang S, Palenik B, Bourne PE. Modern proteomes contain putative imprints of ancient shifts in trace metal geochemistry. Proc Natl Acad Sci. 2006;103:17822-7.

Granger J, Ward BB. Accumulation of nitrogen oxides in copper‐limited cultures of denitrifying bacteria. Limnol Oceanogr. 2003;48:313-8.

Griess, P. Griess Reagent: A Solution of Sulphanilic Acid and Α-Naphthylamine in Acetic Acid Which Gives a Pink Colour on Reaction with the Solution Obtained after Decomposition of Nitrosyl Complexes. Chem. Ber. 1879; 12:427.

Gruber N. The marine nitrogen cycle: overview and challenges. Nitrogen in the marine environment. 2008;2:1-50.

Gustafsson, J.P. Visual MINTEQ ver. 3.0; http://www2.lwr.kth. se/English/OurSoftware/vminteq/index htm. 2010.

Hogle SL, Thrash JC, Dupont CL, Barbeau KA. Trace metal acquisition by marine heterotrophic bacterioplankton with contrasting trophic strategies. Appl Environ Microbiol. 2016;82:1613-24.

Jiang D, Khunjar WO, Wett B, Murthy SN, Chandran K. Characterizing the metabolic trade-off in Nitrosomonas europaea in response to changes in inorganic carbon supply. Environ. Sci. Technol. 2015;49:2523-31.

Jing H, et al. Geographic Distribution of Ammonia-Oxidizing Archaea along the Kuril Islands in the Western Subarctic Pacific. Front. Microbiol.2017; 8:1247.

Kerou M, et al. Proteomics and comparative genomics of Nitrososphaera viennensis reveal the core genome and adaptations of archaeal ammonia oxidizers. Proc Natl Acad Sci U S A. 2016;113:E7937-46.

Kim JW, Price NM. The influence of light on copper‐limited growth of an oceanic diatom, Thalassiosira oceanica (Coscinodiscophyceae). J. Phycol. 2017;53:938-50.

Klotz MG, et al. Complete genome sequence of the marine, chemolithoautotrophic, ammonia-oxidizing bacterium Nitrosococcus oceani ATCC 19707. Appl Environ Microbiol. 2006;72:6299-315.

Kranzler C, Lis H, Shaked Y, Keren N. The role of reduction in iron uptake processes in a unicellular, planktonic cyanobacterium. Environ Microbiol. 2011;13:2990-9.

Leininger S, *et al* Archaea predominate among ammonia-oxidizing prokaryotes in soils. Nature. 2006;442: 806-809.

Lis H, Shaked Y, Kranzler C, Keren N, Morel FM. Iron bioavailability to phytoplankton: an empirical approach. ISME J. 2015;9:1003-13.

Lopez JS, Lee L, Mackey KR. The toxicity of copper to Crocosphaera watsonii and other marine phytoplankton: a systematic review. Front Mar Sci. 2019;5:511.

Maldonado MT, Price NM. Reduction and transport of organically bound iron by Thalassiosira oceanica (Bacillariophyceae). J Phycol. 2001;37:298–309.

Maldonado MT, Strzepek RF, Sander S, Boyd PW. Acquisition of iron bound to strong organic complexes, with different Fe binding groups and photochemical reactivities, by plankton communities in Fe‐limited subantarctic waters. Global Biogeochem. 2005;19.

Marchler-Bauer A, et al. CDD: NCBI's conserved domain database. Nucleic acids Res. 2015;43:D222-6.

Pellitteri-Hahn MC, Halligan BD, Scalf M, Smith L, Hickey WJ. Quantitative proteomic analysis of the chemolithoautotrophic bacterium Nitrosomonas europaea: comparison of growing-and energy-starved cells. J. Proteom. 2011 Apr 1;74:411-9.

Price NM, et al. Preparation and chemistry of the artificial algal culture medium Aquil. Biological oceanography. 1989;6:443-61.

Qin W, et al. Stress response of a marine ammonia-oxidizing archaeon informs physiological status of environmental populations. ISME J. 2018;12:508-19.

Roy EG, Wells ML, King DW. Persistence of iron (II) in surface waters of the western subarctic Pacific. Limnology and Oceanography. 2008;53:89-98.

Santoro AE, et al. Genomic and proteomic characterization of “Candidatus Nitrosopelagicus brevis”: an ammonia-oxidizing archaeon from the open ocean. Proc Natl Acad Sci U S A. 2015;112:1173-8.

Shafiee RT, Snow JT, Zhang Q, Rickaby REM. Iron requirements and uptake strategies of the globally abundant marine ammonia-oxidising archaeon, Nitrosopumilus maritimus SCM1. ISME J. 2019;13:2295-305.

Sunda WG, Huntsman SA. Regulation of copper concentration in the oceanic nutricline by phytoplankton uptake and regeneration cycles. Limnol Oceanogr. 1995;40:132-7.

Tolar BB, et al. Contribution of ammonia oxidation to chemoautotrophy in Antarctic coastal waters. The ISME J. 2016; 10, 2605-2619.

Vissers EW, et al. Temporal and spatial coexistence of archaeal and bacterial amoA genes and gene transcripts in lake Lucerne. Archaea. 2013.

Waterhouse AM, et al. Jalview Version 2- a multiple sequence alignment editor and analysis workbench. Bioinformatics.2009; 25:1189-1191.

Zhang Q, et al. Direct measurement of multi-elements in high matrix samples with a flow injection ICP-MS: application to the extended Emiliania huxleyi Redfield ratio. J Anal At Spectrom. 2018;33:1196-208.

Zorz JK, Kozlowski JA, Stein LY, Strous M, Kleiner M. Comparative proteomics of three species of ammonia-oxidizing bacteria. Front Microbiol. 2018 May 14;9:938.
